# Livestock-Associated Methicillin Resistant *Staphylococcus aureus* (LA-MRSA) Clonal Complex (CC) 398 Isolated from UK Animals belong to European Lineages

**DOI:** 10.3389/fmicb.2016.01741

**Published:** 2016-11-09

**Authors:** Meenaxi Sharma, Javier Nunez-Garcia, Angela M. Kearns, Michel Doumith, Patrick R. Butaye, M. Angeles Argudín, Angela Lahuerta-Marin, Bruno Pichon, Manal AbuOun, Jon Rogers, Richard J. Ellis, Christopher Teale, Muna F. Anjum

**Affiliations:** ^1^Department of Bacteriology, Animal and Plant Health AgencySurrey, UK; ^2^Surveillance and Laboratory Services, Animal and Plant Health AgencySurrey, UK; ^3^National Infection Service, Public Health EnglandLondon, UK; ^4^Faculty of Veterinary Medicine, University of GhentMerelbeke, Belgium; ^5^Department of Biomedical Sciences, School of Veterinary Medicine, Ross UniversityBasseterre, Saint Kitts and Nevis; ^6^Department of Microbiology, National Reference Centre–Staphylococcus aureus, Hôpital Erasme, Université Libre de BruxellesBrussels, Belgium; ^7^Agri-Food and Biosciences InstituteBelfast, UK; ^8^Veterinary and Technical Services, Animal and Plant Health AgencySurrey, UK

**Keywords:** methicillin resistant *Staphylococcus aureus* in animals, whole genome sequencing, UK CC398s, zinc and cadmium resistance, avian prophage genes

## Abstract

In recent years, there has been an increase in the number of livestock-associated methicillin resistant *Staphylococcus aureus* (LA-MRSA) clonal complex (CC) 398 recovered from *S. aureus* isolated animals in the UK. To determine possible origins of 12 LA-MRSA CC398 isolates collected after screening more than a thousand *S. aureus* animal isolates from the UK between 2013 and 2015, whole genome sequences (WGS) of CC398 European, including UK, and non-European isolates from diverse animal hosts were compared. Phylogenetic reconstruction applied to WGS data to assess genetic relatedness of all 89 isolates, clustered the 12 UK CC398 LA-MRSA within the European sub-lineages, although on different nodes; implicating multiple independent incursions into the UK, as opposed to a single introduction followed by clonal expansion. Three UK isolates from healthy pigs and one from turkey clustered within the cassette chromosome recombinases *ccr C S. aureus* protein A (*spa*)-type t011 European sub-lineage and three UK isolates from horses within the *ccrA2B2* t011 European sub-lineage. The remaining UK isolates, mostly from pigs, clustered within the t034 European lineage. Presence of virulence, antimicrobial (AMR), heavy metal (HMR), and disinfectant (DR) resistance genes were determined using an in-house pipeline. Most, including UK isolates, harbored resistance genes to ≥3 antimicrobial classes in addition to β-lactams. HMR genes were detected in most European *ccrC* positive isolates, with >80% harboring *czrC*, encoding zinc and cadmium resistance; in contrast ~60% *ccrC* isolates within non-European lineages and 6% *ccrA2B2* isolates showed this characteristic. The UK turkey MRSA isolate did not harbor φAVβ avian prophage genes (SAAV_2008 and SAAV_2009) present in US MSSA isolates from turkey and pigs. Absence of some of the major human-associated MRSA toxigenic and virulence genes in the UK LA-MRSA animal isolates was not unexpected. Therefore, we can conclude that the 12 UK LA-MRSA isolates collected in the past 2 years most likely represent separate incursions into the UK from other European countries. The presence of zinc and cadmium resistance in all nine food animal isolates (pig and poultry), which was absent from the 3 horse isolates may suggest heavy metal use/exposure has a possible role in selection of some MRSA.

## Introduction

Methicillin-resistant *Staphylococcus aureus* (MRSA) is an opportunistic pathogen able to colonize humans, companion animals and livestock. MRSA in livestock was first reported in 1972 from cases of bovine mastitis, in Belgium where MRSA was found to originate from humans (Devriese et al., 1972). Since then MRSA in animals has been reported infrequently, mostly in companion animals, again originating from humans (Crombé et al., 2013). In 2005 (Voss et al., 2005) identified a new lineage of MRSA, sequence type (ST) 398 grouping within clonal complex (CC) 398 (http://saureus.mlst.net) associated with livestock and able to colonize humans. Following this, livestock associated (LA) MRSA; namely CC398 has been reported globally, in horses, cattle and poultry (Butaye et al., 2016).

In Europe, LA-MRSA has been widely reported from various countries in pigs, poultry and cattle (Butaye et al., 2016), with infrequent reports from the UK. The number of cases of LA-MRSA in farm animals in the UK is gradually increasing with recent reports from, horses, cattle, poultry, pigs, and pork meat products (Loeffler et al., 2009; Paterson et al., 2014). This increase in the number of cases suggests the gradual emergence and change in epidemiology of LA-MRSA among UK livestock. LA-MRSA has been shown to transfer from animals and humans by direct exposure to livestock (Voss et al., 2005). Colonization of LA-MRSA in humans is rare and tends to be asymptomatic and usually transient (Pletinckx et al., 2013). However, should an infection in an immunocompromised individual arise it may be difficult to treat with routine antibiotics, such as penicillin. It is therefore it is important to establish the virulence and antimicrobial potential of these pathogens currently emerging in the UK to ensure antibiotcs are still effective, if treatment is required.

Molecular genotyping methods have long been used to understand the genetic background of *S. aureus* strains, these have included; DNA microarray for detection of resistance and virulence genes; *spa* typing which looks at variable repeats within the *S. aureus* protein A (*spa*); multilocus sequence typing (MLST) of housekeeping genes and classification of the mobile genetic element *Staphylococcal* cassette chromosome (SCC*mec*), the defining feature of MRSA (Stefani et al., 2012). Several types of SCC*mec* have been described by the International Working Group on the Classification of Staphylococcal cassette Chromosome (2009), the various SCC*mec* types can be defined according to the combination of their *ccr* and *mec* complexes.

Although, the principles behind the typing methods described above remain useful for characterization and differentiation of MRSA isolates; employing these methods can be laborious and time consuming. Advances in comparative genomics using whole genome sequencing (WGS), allows for a more rapid method for identifying genetic determinants and for studying phylogenetic relationships between groups of isolates based on the core genome rather than a finite panel of selected genes.

In this study we compared whole genomes sequences (WGS) of UK LA-MRSA isolates collected between 2013 and 2015, to LA-MRSA animal isolates from European and non-European countries. All isolates looked at in this study belonged to the CC398 lineage. UK LA-MRSA livestock isolates selected for WGS were identified from a panel of more than 1000 *S. aureus* strains recovered from pig caeca collected from abattoir and from samples submitted for routine diagnostic testing (Loeffler et al., 2009; Anonymous, 2016) during this period. WGS of methicillin-susceptible *S. aureus* (MSSA) CC398 isolates from animals were also included as comparators to the MRSA isolates as it has been speculated that MRSA CC398 evolved from MSSA CC398 following acquisition of the SCC*mec* element (Vandendriessche et al., 2014).

The aims of the study were to establish the phylogenetic relationship between LA-MRSA CC398 recovered in the UK and those circulating in other countries, and to identify similarities/differences in the carriage of: antimicrobial resistance, heavy metal resistance, disinfectant resistance and virulence genes, to provide insight into the emergence, evolution and possible adaptation of LA-MRSA in livestock in the UK.

## Materials and methods

### Bacterial strains

**Figure 1:**
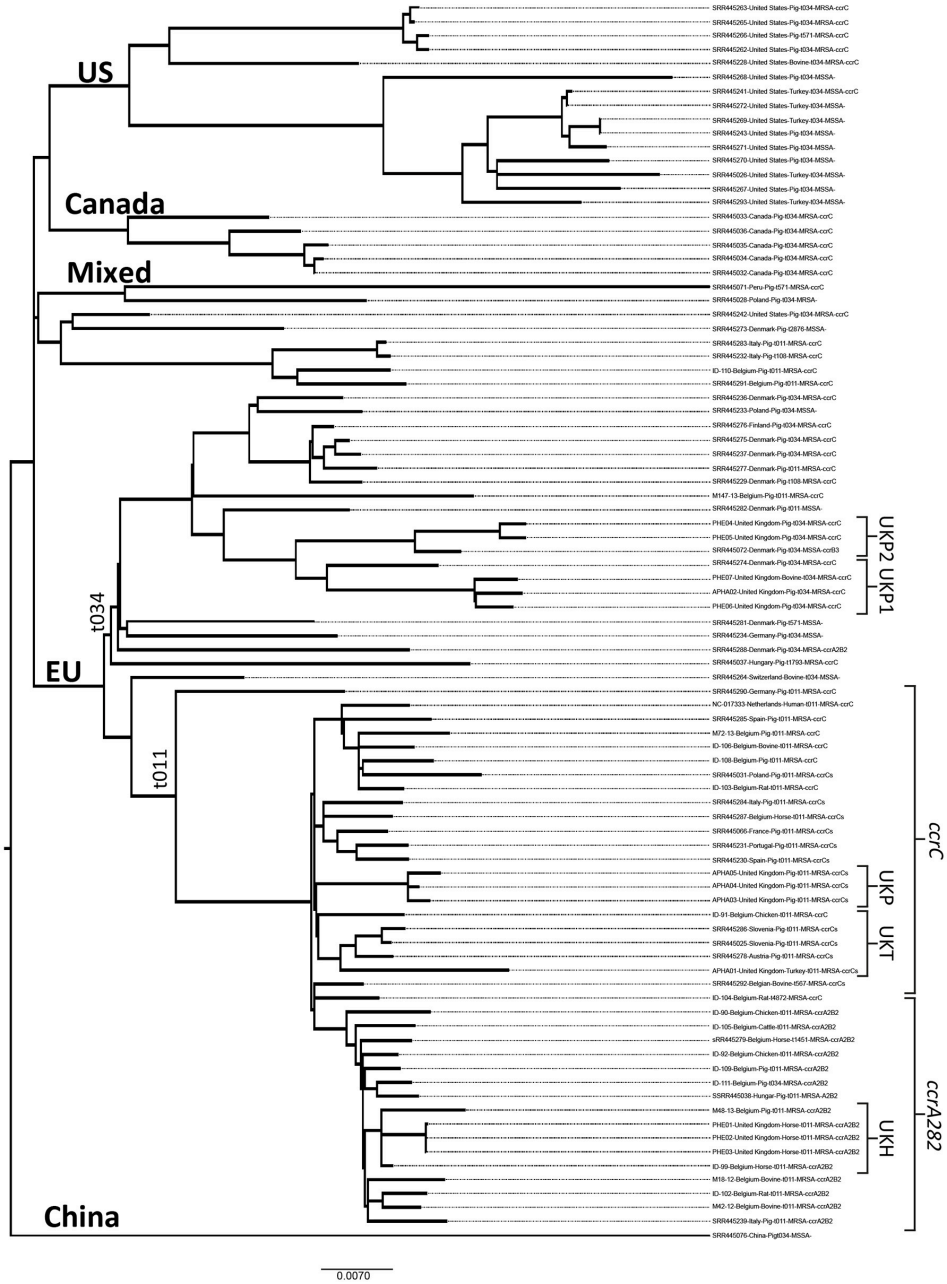
**A maximum-likelihood phylogenetic tree of LA-MRSA CC398 animal isolates calculated in RaxML**. The largely European and non-European lineages, as well as the dominant *spa-*types and *ccr*-types are shown. The following information is also given for each isolate: name/reference, country of origin, host animal, MRSA/MSSA, *spa-*type, and *ccr*-type.

All 89 isolates used in this study belong to CC398 (Table S1). Eighty eight isolates were from various animal species (Figure 1) and the remaining isolate is a commonly used reference strain (SO385) (GenBank NC_017333) of human origin (Table S1).

UK isolates (*n* = 12) were obtained from livestock (horses, poultry, and pigs), reported by the Public Health England (PHE), the Animal and Plant Health Agency (APHA) and the Agri-food and Biosciences Institute (AFBI) (Loeffler et al., 2009; Hall et al., 2015; Anonymous, 2016). Three pig isolates were recovered from caeca collected in a research surveillance study during 2014–15 from 57 healthy pig herds across England at APHA. The remainder of isolates were from *S. aureus* isolated from routine veterinary (pig, poultry, and cattle) diagnostic submissions which were screened for MRSA; this included 586 *S. aureus* isolates screened at AFBI and 609 *S. aureus* isolates screened at APHA between 2013 and 2015. The 3 isolates from two horses were obtained as described by Loeffler et al. (2009).

Diagnostic or caecal material was plated directly onto MacConkey agar (Oxoid Limited) which was incubated overnight at 37°C. Purified suspect *Staphylococcus* colonies were screened by Api Rapidec® Staph (Biomérieux), for species identification. *S. aureus* colonies were susceptibility tested for cefoxitin and a latex agglutination test was performed for the detection of penicillin binding protein (PBP2') (Oxoid Limited). PCR confirmation of positive colonies was carried out as described in the MRSA baseline survey conducted in 2008 by EFSA (2009).

To provide a more extensive representation of European strains, 18 isolates originating from horses, pigs, poultry and rats previously studied (Jamrozy et al., 2012; Nemeghaire et al., 2014; Peeters et al., 2015) were supplied by the Veterinary and Agrochemical Research center (Brussels, Belgium) and the University of Ghent (Ghent, Belgium). The remaining CC398 strains (20 MSSA and 38 MRSA) were from whole genome sequences deposited in the NCBI database (http://www.ncbi.nlm.nih.gov/) from Price et al. (2012), these included European and non-European isolates.

### Antimicrobial susceptibility data

Phenotypic sensitivity data used in this study were available for 28 UK and Belgium isolates. Antimicrobial susceptibility testing was performed and interpreted for the 5 APHA isolates using disc diffusion following BSAC guidelines (http://www.bsac.org.uk/) of the following antimicrobials (disc concentrations): amoxicillin/clavulanate (3 μg); ciprofloxacin (1 μg); cefoxitin (10 μg); erythromycin (5 μg); clindamycin (10 μg); ampicillin (10 μg); penicillin (1 μg); tetracycline (10 μg); trimethoprim/sulphonamide (25 μg). Antimicrobial susceptibility testing for the 7 PHE isolates was performed at PHE and AFB1, by agar dilution method as described Andrews (2001) and disk diffusion method in accordance with the Clinical and Laboratory Standards Institute (CLSI) guidelines (CLSI, 2013a,b), respectively. Antimicrobial susceptibility data from MIC testing was available for 16 Belgian isolates for β-lactams, tetracycline, clindamycin, trimethoprim, aminoglycoside, erythromycin, chloramphenicol, and ciprofloxacin (Jamrozy et al., 2012; Nemeghaire et al., 2014; Peeters et al., 2015).

### Whole genome sequencing

Isolates from Belgium and APHA were sequenced at APHA as described by Anjum et al. (2016). Isolates from PHE and AFBI were sequenced at PHE Genomic Service Unit on Illumina HiSeq platform using Nextera XT library preparations from DNA extracted on QIAsymphony instrument—DNA DSP mini kit. The complete genome of a CC398, *spa* type t011 strain, SO385 (GenBank NC_017333) isolated from a human endocarditis case was used as a reference genome. Sequencing data for 30 isolates from this study have been deposited in the European Nucleotide Archive (ENA) under study PRJEB14251, http://www.ebi.ac.uk/ena/data/view/PRJEB14251.

### Genetic profiling

An in-house software was used to detect virulence (VIR), antimicrobial (AMR), heavy metal (HMR) and disinfectant (DR) resistance genes, as well as *ccr* complex genes and their variants, the avian prophage genes, and chromosomal mutations associated with resistance; these were the reference genes for which sequences were downloaded from NCBI (Table S1; http://www.ncbi.nlm.nih.gov). The in-house software included SMALT (http://www.sanger.ac.uk/science/tools/smalt-0) which mapped reads onto a FASTA-database containing the reference gene sequences. The criteria for determining gene presence was set at ≥95% identity match between the query gene mapped against the reference sequence with a mean coverage of 10-fold with no gaps or non-calls. For the detection of chromosomal mutations associated with antimicrobial resistance, non-synonymous SNPs (ns-SNPs) were identified in query sequences by mapping sequences to reference gene(s) within the same species. SNPs resulting in amino acid differences in positions that have been associated with an AMR phenotype for the following genes were reported: *gyrA, glrA, rpoB, ileS*, and *dfrB* (Table S1).

### Phylogenetic tree generation

Reads from each genome were mapped to the reference genome (NC_017333.1) using BWA (version 0.7.9a) the resulting SAM file was converted to BAM with Samtools (version 1.1). Single nucleotide polymorphisms (SNPs) were called using the Genome Analysis Toolkit 2 (GATK2) and filtered based on the depth of coverage (DP > 5), the ratio of unfiltered reads that support reported alleles were then compared to the reference (AD > 0.8) and the mapping quality determined (MQ > 30). SNPs filtered out using these metrics, including heterozygotes were designated “N.” SNPs identified from each genome were combined to generate a single multiple alignment file, with the maximum proportion of Ns accepted at any position of the alignment set to <20%. A maximum likelihood phylogenetic tree was constructed using RAxML (Stamatakis, 2014) with 500 bootstrap iterations to infer phylogenetic relationship between isolates.

## Results

### Profiling of CC398 isolates using WGS

We compared CC398 isolates collected from UK livestock to animal isolates from other European and non-European countries using WGS. The 89 isolates used in this study represented 9 different *spa* types (t011, t034, t108, t567, t571, t1451, t1793, t2370, and t4872), all belonging to CC398. The most frequently observed *spa* types observed were t034 and t011 (84%), commonly associated with CC398 LA-MRSA. The SNP based maximum-likelihood phylogenetic tree generated in RaxML is shown in Figure 1. There was some evidence of phylogeographic patterns with a majority of European isolates clustering together forming a separate lineage to the non-European isolates. The major lineages and most minor lineages were also dominated by specific *ccr* gene differences with some evidence of *spa* type differentiating within sub-lineages.

All 89 isolates carried genes encoding tetracycline resistance. Approximately 98% encoded resistance to β –lactams and 74% of isolates harbored genes encoding resistance to ≥3 antimicrobial classes (Table 1). Antimicrobial sensitivity data for 28 isolates, including all UK isolates and 16 Belgian isolates, showed the following genotypic and phenotypic associations: 100% for β-lactams, tetracycline, clindamycin; 96% for trimethoprim, erythromycin, fluoroquinolone; 87.5% for phenicols and 75% for aminoglycoside (Table S1), validating the use of WGS to predict antimicrobial resistance for most resistance genes and indicating genes within some antimicrobial classes (phenicols and aminoglycosides) were not fully represented in our reference gene database.

**Table 1:** **The genotypic profiles from WGS of 89 CC398 isolates, including a reference strain**.

**Branch Name**	**Strain Information**		**Gene Group/Number of genes conferring resistance**															
			**Cassette chromosome recombinase (*ccr*) gene complex**				**Zinc and Cadmium**	**Cadmium**	**Fluoroquinolone (mutation)**	**B-lactam**	**Tetracycline**	**Aminoglycoside**	**Chloramphenicol**	**Trimethoprim**	**Macrolide and Lincosamide/ virginamycin**	**Streptogramin A**	**Disinfectant**	**Avian Prophage**
	**Strain Name**	***spa* type**	***ccrA2***	***ccrB2***	***ccrB3***	***ccrC***	***czrC***	***cadD***	**2**	**2**	**3**	**8**	**6**	**5**	**7**	**1**	**3**	**2**
US	SRR445263	t034	0	0	0	1	1	0	WT	2	2	2	0	0	3	0	0	0
	SRR445265	t034	0	0	0	1	1	0	WT	2	2	2	0	0	3	0	0	0
	SRR445266	t571	0	0	0	1	1	0	WT	2	2	1	0	0	1	0	0	0
	SRR445262	t034	0	0	0	1	1	0	WT	2	2	1	0	0	3	0	0	0
	SRR445228	t034	1	1	0	0	0	0	WT	0	1	0	0	0	0	0	0	0
	SRR445268	t034	0	0	0	0	0	0	WT	1	1	1	0	0	0	0	0	2
	SRR445241	t034	0	0	0	1	0	0	WT	1	1	0	0	0	1	0	0	2
	SRR445272	t034	0	0	0	0	0	1	WT	1	1	0	0	0	2	0	0	2
	SRR445269	t034	0	0	0	0	0	0	WT	1	1	0	0	0	0	0	0	2
	SRR445243	t034	0	0	0	0	0	0	WT	1	1	0	1	0	2	0	0	0
	SRR445271	t034	0	0	0	0	0	0	WT	1	1	0	0	0	0	0	0	2
	SRR445270	t034	0	0	0	0	0	0	WT	1	1	0	0	0	0	0	0	2
	SRR445026	t034	0	0	0	0	0	0	WT	1	2	0	0	0	0	0	0	2
	SRR445267	t034	0	0	0	0	0	0	WT	1	1	0	0	0	0	0	0	2
	SRR445293	t034	0	0	0	0	0	0	WT	1	2	0	0	0	0	0	0	2
Canada	SRR445033	t034	0	0	0	1	0	0	WT	2	1	1	0	0	2	0	1	0
	SRR445036	t034	0	0	0	1	0	0	WT	2	1	3	0	0	1	0	1	0
	SRR445035	t034	0	0	0	1	0	0	WT	2	1	0	0	0	1	0	1	0
	SRR445034	t034	1	0	0	1	1	1	WT	1	2	3	0	0	1	0	1	0
	SRR445032	t034	0	0	0	1	1	0	WT	1	2	0	0	0	1	0	1	0
Mixed	SRR445071	t571	1	0	0	1	0	1	MT	2	2	1	1	2	2	1	0	0
	SRR445028	t034	0	0	0	0	0	1	MT	2	1	2	0	0	1	0	0	0
	SRR445242	t034	0	0	0	1	1	0	WT	1	1	1	0	0	1	0	0	0
	SRR445273	t2876	0	0	0	0	0	0	WT	1	1	0	0	1	1	0	0	0
	SRR445283	t011	0	0	0	1	1	1	MT	2	1	2	0	0	1	0	0	0
	SRR445232	t108	0	0	0	1	1	0	MT	2	1	1	0	0	1	0	0	0
	ID-110	t011	0	0	0	1	0	0	WT	2	1	0	0	0	1	0	0	0
	SRR445291	t011	0	0	0	1	0	0	U	2	1	2	0	0	0	0	0	0
EU t034	SRR445236	t034	0	0	0	1	0	1	WT	2	2	2	1	1	1	0	0	0
	SRR445233	t034	0	0	0	0	0	0	WT	1	1	1	3	1	1	0	0	0
	SRR445276	t034	1	1	0	1	1	0	WT	2	2	4	1	2	2	0	3	0
	SRR445275	t034	0	0	0	1	1	0	WT	2	2	0	1	1	1	0	0	0
	SRR445237	t034	0	0	0	1	1	0	WT	2	2	2	0	1	1	0	0	0
	SRR445277	t034	0	0	0	1	1	0	WT	2	2	1	0	1	1	0	0	0
	SRR445229	t108	0	0	0	1	1	0	WT	2	1	1	0	1	0	0	0	0
	M147-13	t011	0	0	0	1	1	0	MT	1	2	1	1	0	1	0	0	0
	SRR445282	t011	0	0	0	0	0	0	U	1	1	0	0	0	0	0	0	0
	PHE04	t034	0	0	0	1	1	1	MT	2	2	0	0	2	0	0	0	0
	PHE05	t034	0	0	0	1	1	1	MT	2	2	1	0	2	0	0	0	0
	SRR445072	t034	0	1	1	0	1	1	MT	1	1	0	0	2	1	0	0	0
	SRR445274	t034	0	0	0	1	0	1	WT	1	2	1	1	2	1	0	1	0
	PHE07	t034	0	0	0	1	1	0	WT	2	2	0	0	2	2	0	0	0
	APHA02	t034	0	0	0	1	1	0	WT	2	2	1	0	3	1	0	0	0
	PHE06	t034	0	0	0	1	1	0	WT	2	2	0	0	2	1	0	0	0
	SRR445281	t571	0	0	0	0	0	1	U	0	1	0	0	2	2	0	0	0
	SRR445234	t034	0	0	0	0	0	0	MT	1	1	1	0	0	1	0	0	0
	SRR445288	t034	1	1	0	0	0	0	U	1	0	0	0	0	0	0	0	0
	SRR445037	t1793	0	0	0	1	1	0	MT	2	2	1	0	0	3	0	0	0
EU t011	SRR445264	t034	0	0	0	0	0	0	WT	0	1	0	0	0	0	0	0	0
	SRR445290	t011	0	0	0	1	1	0	U	2	1	1	0	1	0	0	0	0
	NC-017333	t011	0	0	0	1	1	0	MT	2	1	0	0	0	0	0	0	0
	SRR445285	t011	0	0	0	1	U	0	MT	2	0	0	0	0	1	0	0	0
	M72-13	t011	0	0	0	1	1	0	MT	2	2	1	0	3	2	0	0	0
	ID-106	t011	0	0	0	1	1	0	MT	2	2	2	0	3	1	0	0	0
	ID-108	t011	0	0	0	1	1	0	MT	2	2	1	0	3	3	0	0	0
	SRR445031	t011	0	0	0	1	1	0	MT	2	2	1	0	2	1	0	0	0
	ID-103	t011	0	0	0	1	1	0	MT	2	2	1	0	3	3	0	0	0
	SRR445284	t011	0	0	0	1	1	0	U	2	2	0	0	0	0	0	0	0
	SRR445287	t011	0	0	0	1	U	0	U	2	0	0	0	0	0	0	0	0
	SRR445066	t011	0	0	0	1	1	0	WT	2	2	0	0	0	1	0	0	0
	SRR445231	t011	0	0	0	1	1	0	WT	2	2	1	0	0	1	0	0	0
	SRR445230	t011	0	0	0	1	1	0	WT	2	2	0	0	0	2	0	0	0
	APHA05	t011	0	0	0	1	1	0	MT	2	2	0	0	0	2	0	0	0
	APHA04	t011	0	0	0	1	1	0	MT	2	2	0	0	0	1	0	0	0
	APHA03	t011	0	0	0	1	1	0	MT	2	2	0	0	0	1	0	0	0
	ID-91	t011	0	0	0	1	1	0	WT	2	2	1	1	0	2	0	0	0
	SRR445286	t011	0	0	0	1	1	1	U	2	3	0	0	1	0	0	0	0
	SRR445025	t011	0	0	0	1	1	1	MT	2	3	1	0	1	0	0	0	0
	SRR445278	t011	0	0	0	1	1	0	MT	2	2	1	0	0	2	0	0	0
	APHA01	t011	0	0	0	1	1	0	WT	2	2	0	0	0	0	0	0	0
	SRR445292	t567	0	0	0	1	1	0	U	1	0	2	0	0	0	0	0	0
	ID-104	t4872	0	0	0	1	0	1	WT	2	2	3	0	0	3	0	0	0
	ID-90	t011	1	1	0	0	0	1	WT	2	2	2	0	2	2	0	0	0
	ID-105	t011	1	1	0	0	0	1	WT	2	2	1	0	3	3	0	0	0
	SRR445279	t1451	1	1	0	0	0	1	WT	2	3	2	0	1	3	0	0	0
	ID-92	t011	1	1	0	0	0	1	WT	2	2	3	0	2	3	0	0	0
	ID-109	t011	1	1	0	0	0	0	WT	2	1	2	0	2	1	0	0	0
	ID-111	t034	1	1	0	0	0	0	WT	2	1	2	0	3	1	0	0	0
	SRR445038	t011	1	1	0	0	1	0	WT	2	2	1	0	2	3	0	0	0
	M48-13	t011	1	1	0	0	0	0	WT	2	1	1	0	3	0	0	0	0
	PHE01	t011	1	1	0	0	0	0	WT	2	1	2	0	2	0	0	0	0
	PHE02	t011	1	1	0	0	0	0	WT	2	1	2	0	2	0	0	0	0
	PHE03	t011	1	1	0	0	0	0	WT	2	1	2	0	2	0	0	0	0
	ID-99	t011	1	1	0	0	0	0	WT	2	1	2	0	3	0	0	0	0
	M18-12	t011	1	1	0	0	0	1	WT	2	2	3	0	3	4	0	0	0
	ID-102	t011	1	1	0	0	0	0	WT	2	1	2	0	3	3	0	0	0
	M42-12	t011	1	1	0	0	0	1	WT	2	2	2	0	3	2	0	0	0
	SRR445239	t011	1	1	0	0	0	1	WT	2	2	2	1	1	1	0	3	0
China	SRR445076	t034	0	0	0	0	0	0	MT	1	2	1	1	0	2	0	0	0

*Only genes present in our panel of isolates are shown. Genes belonging to the same antimicrobial class have been grouped: β-lactams (blaZ, mecA); aminoglycosides (aac(6′)-aph(2″), aadD-aph(4)-Ia, aadE-ant(6)-Ia, ant9-Ia, aphA3-aph(3′)-III, strA, spd, and spw); trimethoprim (dfrA, dfrC, dfrD, dfrG, and dfrK), macrolide and lincosamides/virginamycin (ermA, ermB, ermC, ermT, ermY, lnuA, and vga), tetracycline (tetM, tetL, and tetK), streptothricin (sat), phenicols [cat (4 variants), cfr and fexA] and fluoroquinolone (grlA and gyrA mutations). Genes encoding resistance to disinfectants (qacA, qacB, and qacC-smr) or the avian prophage genes (SAAV_2008, SAAV_2009) have also been grouped. Genes encoding zinc and cadmium resistance are given. For detail of each genotypic profile see Table S1. WT, wild type; MT, SNP mutant; U, unknown (data below our threshold)*.

Nine percent of isolates harbored genes which have been associated with resistance to quaternary ammonium compounds (*qacA, qacB*, and *qacC-smr*) and 64% carried genes conferring resistance to ≥1 heavy metals (*czrC, cadD*). The SAAV_2008 and SAAV_2009 genes associated with φAVβ avian prophage were detected in 9 non-UK strains, isolated from turkey and pigs (Table 1).

### Characteristics of European isolates

The European (EU) lineage divided into two main sub-lineages, based on *spa* type, designated EU t011 and EU t034 (Figure 1).

### EU t0ll lineage

The largest European sub-lineage, EU t011, comprised entirely of EU LA-MRSA strains of *spa* type t011, these were from a broad range of hosts including rats, cattle, pigs, poultry and horses. Two further sub-lineages were distinguishable by their *ccr* genes (Figure 1); one group harbored *ccr*C (associated with SCC*mec*V), the second carried both *ccrA2* and *ccrB2* (associated with SCC*mec*IV). Lineage EU t011 isolates harbored between 3 and 11 AMR genes/mutations conferring resistance to the following antimicrobials: β-lactams, tetracyclines, aminoglycosides, chloramphenicol, trimethoprim, macrolides, and lincosamides (Table 1).

#### Sub-lineage *ccrC* within EU t011

Several of the UK isolates were present within sub-lineage EU t011 carrying *ccrC*, these included a turkey strain (APHA01) isolated from the lung of a turkey carcass in England and three pig strains isolated from the caeca of healthy pigs in England (APHA 03, 04, and 05). APHA01 presented closest homology to 3 pig isolates from Slovenia (SRR445286 and SRR445025) and Austria (SRR445278), and a chicken isolate (ID-91) from Belgium, forming a paraphyletic group (UKT) within the EU t011 cluster (Figure 1). Carriage of AMR genes varied between strains in UKT; with APHA01 encoding resistance only to β-lactams and tetracycline while the Belgian, (ID-91), Slovenian (SRR445286, SRR445025, and Austrian (SRR445278) strains carried additional (1–4) AMR genes, conferring resistance to aminoglycosides, chloramphenicol, macrolides and trimethoprim (Table 1). Although, APHA01 was isolated from turkey it did not harbor the avian prophage genes (SAAV_2008 and SAAV_2009).

The UK pig isolates (APHA 03, 04, and 05) formed a monophyletic group (UKP) which showed close homology to the UKT sub-group. All 3 UK isolates within UKP presented β-lactam and tetracycline resistance genes, similar to APHA01. However, in addition to this, the UKP group also harbored *vgaA* (macrolide and lincosamide/virginamide resistance) and fluoroquinolone resistance due to a mutation in *grlA*. One isolate, APHA05, harbored *ermC*, conferring to the macrolide-lincosamide and streptogramin (Table S1). Nineteen of 22 *ccrC* isolates, including the UK isolates, harbored HMR gene *czrC* (zinc resistance). Data for 3 of the 22 *ccrC* isolates did not give a definitive result with respect to the *czrC* gene; two of the isolates fell below the set quality threshold of ≥95% and for one isolate the *czrC* gene was absent. Two isolates from Slovenia and a Belgian isolate harbored HMR, *cadD* encoding cadmium resistance (Table 1).

#### Sub-lineage *ccrA2B2* within EU t011

UK horse isolates (PHE01, 02, and 03) were found to be most closely related to 2 Belgian horse (ID-99) strains and a pig strain (M48-13), called UKH (Figure 1). These strains carried *blaZ, mecA, tetM, aac(6')-aph(2”), dfrK* genes encoding resistance to β-lactams, tetracycline, aminoglycoside and trimethoprim, and *strA* (streptomycin resistance). In addition to these genes ID-99 also carried *spd* (spectinomycin resistance; Table S1). Carriage of HMR genes *cadD*, was observed in 6 Belgian isolates. Only 1 isolate (SRR445038) within this sub-lineage harbored *czrC*. None of the UKH group harbored any HMR genes. An isolate from Italy (SRR445239) was found to harbor DR *qac* genes, associated with decreased susceptibility to quaternary ammonium compounds (Table 1; Table S1).

### EU t034 lineage

#### Sub-lineage *ccrC* within EU t034

The majority of isolates within EU t034 carried *ccrC* genes with a restrictive host range of pigs. A limited geographic distribution was observed with all isolates belonging to Europe (Figure 1). A small number of isolates within this lineage were MSSA strains therefore lacked *mecA* and the *ccr* genes. Majority of the isolates within this sub-lineage, carried between 1 and 12 AMR genes/mutations (Table 1). This lineage contained 3 UK isolates; APHA02 was isolated from a skin lesion of a diseased pig in England and the two Northern Ireland strains were recovered from a pig with pneumonia and a cow with bovine mastitis (PHE 06 and PHE 07), hereafter called UKP1 group. In addition to β-lactam resistance UKP1 harbored AMR genes to macrolides/lincosamides and trimethoprim (*lnuB* and *dfrG*). PHE07 also carried *ermC* (macrolide-lincosamide-streptogramin resistance) and APHA02 carried aminoglycoside resistance gene *strA*. The remaining two UK isolates, PHE04, and PHE05 (UKP2) from post-weaning piglet with porcine reproductive and respiratory syndrome and lesions resulting from pneumonia, formed a separate sub-cluster from UKP1, although they belong to the same sub-lineage. The UKP2 isolates clustered more closely with an MSSA pig stain from Denmark (SRR445072). While the Danish strain lacked *mecA*, it harbored genes/mutations encoding β-lactam, trimethoprim and fluoroquinolone resistance as did the UKP2 strains (Table 1).

In addition, 12 of the 14 *ccrC* isolates in this subgroup harbored *czrC* and one isolate without *ccr*C also harbored *czrC*; six isolates encoded *cadD* of which three were *ccrC* positive (Table 1). One isolate (SRR44523) harbored all three *qac* genes, whilst one showed presence only of *qacC*-*smr* (SRR445274).

### Characteristics of non-european isolates

The US, Canada lineages (Figure 1) included isolates that were mostly of *spa* type t034 from pigs, turkeys and cattle, including both LA-MRSA and MSSA strains (Figure 1). The LA-MRSA strains carried the *ccrC* complex. Majority of the isolates in the non-EU lineages harbored a greater diversity of AMR genes compared to the EU and UK isolates, carrying between 2 and 11 genes associated with resistance to: β-lactams, tetracyclines, aminoglycosides, phenicols, trimethoprim, macrolides and lincosamides/virginamycin, streptothricin, and fluoroquinolone (Table 1). Nine *ccrC* isolates harbored HMR, carrying *czrC* genes and *cadD* genes. Three *ccrC* isolates carried only *cadD* genes, two MSSA strains were found that carried *cadD*. All five isolates from Canada harbored the *qacC*-*smr* disinfectant resistance genes and nine MSSA isolates from the US carried the SAAV_2008 and SAAV_2009 prophage genes (Table S1).

A handful of isolates clustered within the “mixed” lineage (Figure 1), which predominately belonged to non-European isolates from Peru, Belgium, Poland, Denmark, US and Italy, and were a mixture of *spa*-types. One individual strain from China did not cluster within any of the lineages.

## Discussion

The antimicrobial resistance and virulence potential of LA-MRSA has been well documented for mainland Europe and other countries globally (Guardabassi et al., 2007; Price et al., 2012). However, there is currently little data published on the pathogenicity, antimicrobial resistance and phylogeny of UK LA-MRSA CC398 directly isolated from animal sources (Ward et al., 2014). It is therefore important to establish how UK LA-MRSA characteristics compare with those circulating in Europe and elsewhere, especially as LA-MRSA isolates have been found to carry genes that confer resistance to antimicrobials used to treat serious veterinary and human infections.

Also, WGS and downstream applications are increasingly being applied to pathotype microorganisms, to provide a view on the epidemiology, phylogeny and microevolution of bacterial pathogens; including MRSA (Fitzgerald, 2012; Price et al., 2012; Tong et al., 2015). Advances in comparative genomics, using WGS, allows for a more rapid and accurate method to determine phylogenetic relationships between groups of isolates compared to more traditional typing and screening methods such as MLST and DNA microarray. For phylogeny, WGS analysis considers nsSNPs of a much larger number of core/common genes (usually several hundred) present in a set of isolates when predicting phylogeny in comparison to typing schemes such as MLST, which only looks at seven house-keeping genes (http://pubmlst.org/saureus/). For detection of virulence and AMR genes, WGS has the potential to probe for the presence of an infinite number of genes, and the ability to identify emerging variants whilst DNA microarrays can only probe a limited number of virulence and AMR determinants (Piccinini et al., 2012). Therefore, in this study, WGS was used to determine the phylogeny and molecular characteristics of 12 LA-MRSA CC398s isolated from animals in the UK after screening more than a thousand *S. aureus*, between 2013 and 2015. As LA-MRSA CC398 is a zoonotic pathogen that has been reported infrequently in the UK, but is more prevalent elsewhere in Europe, WGS data from CC398 isolates from European (including UK) and non-European countries were compared.

Molecular characteristics that were explored in isolates using WGS included AMR, HMR, DR, and virulence. The use of WGS to infer AMR in MRSA is becoming more common place and has been argued to be at least as reliable as phenotypic testing (Aanensen et al., 2016). The results from this study show most LA-MRSA isolates, including several from the UK, harbored multiple AMR genes with the number ranging between 3 and 12, per isolate. In some instances multiple genes encoding resistance to the same antimicrobial class (e.g., macrolides) were present, which was not unexpected. For the subset of isolates with phenotypic data available, there was good correlation (>96%) between the geno- and pheno-types of most antimicrobials compared, except phenicols, which was nevertheless >85% and the aminoglycosides (75%). This suggests there may be genes/chromosomal mutations responsible for the latter phenotypes that were not included in our pipeline, which requires further investigation. The presence of multiple AMR genes in our LA-MRSA isolates raises concern as they add potentially transferable resistances to the bacterial gene pool present in humans and animals that can be passed to other bacteria including other strains of *S. aureus*.

It has been considered that CC398 originated in humans, probably as a MSSA and transferred to animals where it evolved by losing some of the virulence genes associated with human adaptation allowing successful colonization of a broader host range, and acquiring the *mecA* gene (Price et al., 2012). This was followed by acquisition of the SCC*mec* element (Vandendriessche et al., 2014), conferring resistance to β-lactams and also to tetracycline. Tetracycline resistance is attributed to its historic use in veterinary medicine as a growth promoter added to animal feed, a practice now being banned across the EU. Another resistance attributed to its use in animal feed is resistance to zinc (Cavaco et al., 2011). From this study the *mecA* gene was present in all isolates excluding the MSSA strains. Genes encoding tetracycline resistance were ubiquitous; all isolates, except one harbored *tetM*, which is well established in the CC398 livestock clade (Stegger et al., 2013). One MSSA strain conferred tetracycline resistance through *tetK* and *tetL*.

Presence of zinc and cadmium resistance genes was variable in the panel of isolates included in this study. The *czrC* gene confers resistance to both zinc and cadmium, and was present in all the UK CC398s relating to food animals but not in the 3 UK isolates from horses. Although the sample size was limited to 89 isolates, due to lack of WGS data from LA-MRSA CC398 being widely available, our results showed a strong correlation between the SCC*mec*V (associated with *ccrC* complex) and carriage of *czrC*, supporting a report by Cavaco et al. (2010) who concluded that a variant of SCC*mec*V carried *czrC* (designated subtype Vc). Their study also showed 100% correlation between the presence of *czrC* and phenotypic resistance to zinc (Cavaco et al., 2010), therefore it is likely that isolates in our panel harboring *czrC* would show resistance to zinc phenotypically. The majority of *ccrC* isolates from Europe (~86%) showed correlation between presence of *ccrC* and *czrC*, this was greater than in the non-European isolates (~60%). In contrast, only 6% of isolates harboring *ccrA2B2* carried the *czrC* gene. The low incidence *czrC* genes in *ccrA2B2* isolates may impact on the prevalence of this lineage in the future, if environmental selection pressure from heavy metals such as zinc increases or *ccrA2B2* isolates containing *czrC* may become more widespread. Zinc is approved for inclusion in animal feed in some European countries and the selective effects of such inclusion on the occurrence of MRSA have been reported (Moodley et al., 2011) and debated (Burch, 2014), Our results suggest a possible impact on the epidemiology and incidence of different LA-MRSA subtypes, which require further scrutiny. In contrast, only 23% of isolates carried the *cadD* genes conferring resistance to cadmium only, which has been detected as a contaminant in animal feed (Farmer and Farmer, 2000). Genes encoding resistance to quaternary ammonium compounds which are used widely as disinfectants on farms and the food industry were present in just 9% of isolates.

The WGS data were also used to infer phylogeny, which clustered the European isolates within two main lineages, EU t011 and t034 that separated according to *spa* type. EU t011 included the majority of European *spa*-type t011 isolates present in our panel which were from a variety of animal host species i.e., host generalist, including turkeys, pigs, rats and horses. These isolates formed a distinct lineage but there was clear separation of isolates harboring *ccrA2B2*, part of the SCC*mec* IV cassette, from isolates harboring *ccrC*, part of the SCC*mec* V cassette. All seven UK *spa*-type t011 isolates from pig, turkey and horse were within this host generalist lineage. The second European lineage (EU t034) predominantly included *spa*-type t034 isolates from pigs; which was distinct from the non-European t034 isolates that included isolates from pigs but also from turkeys, which indicates radiation and adaptation of *spa* type t034 in different host species. The five remaining UK LA-MRSA CC398 isolates clustered within the EU t034 branch, and included several isolates from diseased pigs and one from a cow with mastitis. In fact out of the six UK MRSA CC398 isolates that were recovered from diseased animals, MRSA was thought to be responsible for clinical disease only in APHA02. APHA02 was recovered from swabs taken from skin lesion of diseased piglets, where subsequent culture yielded profuse growth of haemolytic *S. aureus* colonies. Downstream testing by PCR of suspect colonies showed presence of *mecA* (Hall et al., 2015). The occurrence of UK MRSA isolates of both *spa*-type t011 and t034 in different sub-lineages indicates separate incursions of LA-MRSA CC398 into the UK, rather than expansion of a single MRSA clone into different animal hosts.

It is worth noting that several MSSA isolates clustered within the EU t034 sub-lineage, this could indicate instability and loss of the SCC*mec* V in these isolates or point to multiple independent acquisition events.

It has been proposed that CC398 strains have lost genes associated with virulence in humans as a result of anthroponotic transmission, which has resulted in low level epidemicity in humans (Fitzgerald, 2012). None of the isolates explored in this study carried any human associated virulence genes, in concordance with a previous study (Price et al., 2012) and the above suggestions. However, SAAV_2008 and SAAV_2009 virulence genes associated with the avian adapted φAVβ prophage that have been reported by several authors (Price et al., 2012; Argudin et al., 2013; Abdelbary et al., 2014), were identified in nine MSSA isolates from the US from turkeys and pigs. The avian prophage genes were not detected in any of the UK isolates, including the turkey MRSA isolate (APHA01). Presence of the φAVβ prophage in MSSA but not MRSA in this and other studies (Argudin et al., 2013; Abdelbary et al., 2014) may suggest an association between the φAVβ prophage and MSSA isolates, which requires further investigation.

In conclusion, our data has shown a European origin for the 12 UK CC398 MRSAs from livestock, as demonstrated by the phylogenetic reconstruction performed using WGS data. There are indications from this reconstruction that there have been multiple independent incursions into the UK rather than clonal expansion following a single introduction. To our knowledge this is the first such study which has been performed using WGS data from UK CC398 LA-MRSA livestock isolates. The collection of strains assembled may have some bias as it only includes isolates available to us and WGS from CC398 LA-MRSA of livestock origin available from public archives. In future we hope to repeat the analysis with a larger collection of CC398 isolates from the UK, as well as globally to determine how representative these isolates may be of those that are found in the UK. The number and types of AMR genes present in the UK isolates were similar to other CC398 LA-MRSA of European origin. The virulence genes included in our pipeline were not detected in any of the UK isolates, despite one isolate being associated with disease (APHA02). It is possible that this LA-MRSA isolate harbors virulence determinants that were not included in our pipeline or that other factors predisposed the piglets to an opportunistic staphylococcal infection. Lastly, genes encoding resistance to heavy metals such as zinc and cadmium were present in ~86% of European isolates including the UK CC938 livestock isolates; the degree to which these genes contribute to survival, dissemination and selection of LA-MRSA in livestock is worthy of further study.

We believe this is an important study because it not only indicates the emergence and possible origins of LA-MRSA in UK livestock but its potential to transmit and evolve through different animal host species. It is only by identifying the clonal origin of UK LA-MRSA isolates alongside continual monitoring of genetic traits that appropriate biosecurity and control measures, as well as methods for restricting transmission, can be considered in future.

## Author contributions

Laboratory work – MS, MAA, BP, MAb, JR, and RE. Data analysis and Bioinformatics – JN, AK, MD, BP, and MFA. Manuscript preparation and review – MA, AK, CT, MAA, MS, JN, MD, PB, AL, BP, and RE.

## Funding

This work was funded by the Veterinary Medicines Directorate through grants VM0506 and RDVM0518 to CT and MFA. MAA was supported by a research grant from the Fundación Alfonso Martín Escudero.

### Conflict of interest statement

The authors declare that the research was conducted in the absence of any commercial or financial relationships that could be construed as a potential conflict of interest.

## Supplementary material

Table S1 **The genotypic and phenotypic characterization of 89 CC398 isolates used in this study, including a reference strain and isolates whose WGS were downloaded from NCBI**. For all isolates strain number, country of origin, spa type and phenotypic data, where available, are given. The AMR, HMR, DR, and virulence genes that were present in the isolates and analyzed using an in-house pipeline are also given.

## References

[B1] Aanensen D. M. Feil E. J. Holden M. T. Dordel J. Yeats C. A. Fedosejev A.. (2016). Whole-Genome Sequencing for Routine Pathogen Surveillance in Public Health: a Population Snapshot of Invasive *Staphylococcus aureus* in Europe. mBio 7:e00444-16. 10.1128/mBio.00444-16 27150362 PMC4959656

[B2] Abdelbary M. M. Wittenberg A. Cuny C. Layer F. Kurt K. Wieler L. H.. (2014). Phylogenetic analysis of *Staphylococcus aureus* CC398 reveals a sub-lineage epidemiologically associated with infections in horses. PLoS ONE 9:e88083. 10.1371/journal.pone.0088083 24505386 PMC3913741

[B3] Andrews J. M. (2001). Determination of minimum inhibitory concentrations. J. Antimicrob. Chemother. 48, 5–16. 10.1093/jac/48.suppl_1.5 11420333

[B4] Anjum M. F. Duggett N. A. AbuOun M. Randall L. Nunez-Garcia J. Ellis R. J.. (2016). Colistin resistance in Salmonella and *Escherichia coli* isolates from a pig farm in Great Britain. J. Antimicrob. Chemother. 71, 2306–2313. 10.1093/jac/dkw149 27147305

[B5] Anonymous (2016). Veterinary medicines update. Vet Rec 178, 330–331. 10.1136/vr.i1772 27034295

[B6] Argudín M. A. Cariou N. Salandre O. Le Guennec J. Nemeghaire S. Butaye P. (2013). Genotyping and antimicrobial resistance of *Staphylococcus aureus* isolates from diseased turkeys. Avian. Pathol. 42, 572–580. 10.1080/03079457.2013.854308 24224550

[B7] Burch D. G. (2014). Effects of tetracycline and zinc on selection of methicillin-resistant *Staphylococcus aureus* (MRSA) sequence type 398 in pigs (Moodley et al., 2011). Vet. Microbiol. 173, 398–400. 10.1016/j.vetmic.2014.08.021 25236984

[B8] Butaye P. Argudin M. A. Smith T. C. (2016). Livestock-associated MRSA and its current evolution. Curr. Clin. Micro Rpt. 3, 19–31. 10.1007/s40588-016-0031-9

[B9] Cavaco L. M. Hasman H. Aarestrup F. M. (2011). Zinc resistance of *Staphylococcus aureus* of animal origin is strongly associated with methicillin resistance. Vet. Microbiol. 150, 344–348. 10.1016/j.vetmic.2011.02.014 21411247

[B10] Cavaco L. M. Hasman H. Stegger M. Andersen P. S. Skov R. Fluit A. C.. (2010). Cloning and occurrence of czrC, a gene conferring cadmium and zinc resistance in methicillin-resistant *Staphylococcus aureus* CC398 isolates. Antimicrob. Agents Chemother 54, 3605–3608. 10.1128/AAC.00058-10 20585119 PMC2934997

[B11] Clinical Laboratory Standards Institute (CLSI) (2013a). CLSI Document VET01-A4: Performance Standards for Antimicrobial Disk and Dilution Susceptibility Tests for Bacteria Isolated from Animals, 4th Edn. Wayne, PA: Clinical and Laboratory Standards Institute.

[B12] Clinical Laboratory Standards Institute (CLSI) (2013b). CLSI Document VET01-S2: Performance Standards for Antimicrobial Disk and Dilution Susceptibility Tests for Bacteria Isolated from Animals. Second Informational Supplement. Wayne, PA: Clinical and Laboratory Standards Institute.

[B13] Crombé F. Argudín M. A. Vanderhaeghen W. Hermans K. Haesebrouck F. Butaye P. (2013). Transmission dynamics of methicillin-resistant *Staphylococcus aureus* in Pigs. Front. Microbiol. 4:57. 10.3389/fmicb.2013.00057 23518663 PMC3602589

[B14] Devriese L. A. Van Damme L. R. Fameree L. (1972). Methicillin (cloxacillin)-resistant *Staphylococcus aureus* strains isolated from bovine mastitis cases. Zentralbl. Veterinärmed. 19, 598 – 605. 10.1111/j.1439-0450.1972.tb00439.x 4486473

[B15] EFSA (2009). Analysis of the baseline survey on the prevalence of methicillin-resistant *Staphylococcus aureus* (MRSA) in holdings with breeding pigs, in the EU, 2008 - Part A: MRSA prevalence estimates. EFSA J. 7:1376 10.2903/j.efsa.2009.1376

[B16] Farmer A. A. Farmer A. M. (2000). Concentrations of cadmium, lead and zinc in livestock feed and organs around a metal production centre in eastern Kazakhstan. Sci. Total Environ. 257, 53–60. 10.1016/S0048-9697(00)00497-6 10943902

[B17] Fitzgerald J. R. (2012). Human origin for livestock-associated methicillin-resistant Staphylococcus aureus. MBio 3, e00082–e00012. 10.1128/mBio.00082-12 22511352 PMC3345579

[B18] Guardabassi L. Stegger M. Skov R. (2007). Retrospective detection of methicillin resistant and susceptible *Staphylococcus aureus* ST398 in Danish slaughter pigs. Vet. Microbiol. 122, 384–386. 10.1016/j.vetmic.2007.03.021 17467199

[B19] Hall S. Kearns A. Eckford S. (2015). Livestock-associated MRSA detected in pigs in Great Britain. Vet. Rec. 176, 151–152. 10.1136/vr.h627 25655544

[B20] International Working Group on the Classification of Staphylococcal cassette Chromosome, Elements (2009). Classification of staphylococcal cassette chromosome mec (SCCmec): guidelines for reporting novel SCCmec elements. Antimicrob Agents Chemother 53, 4961–4967. 10.1128/AAC.00579-09 19721075 PMC2786320

[B21] Jamrozy D. M. Fielder M. D. Butaye P. Coldham N. G. (2012). Comparative genotypic and phenotypic characterisation of methicillin-resistant *Staphylococcus aureus* ST398 isolated from animals and humans. PLoS ONE 7:e40458. 10.1371/journal.pone.0040458 22792335 PMC3394705

[B22] Loeffler A. Kearns A. M. Ellington M. J. Smith L. J. Unt V. E. Lindsay J. A.. (2009). First isolation of MRSA ST398 from UK animals: a new challenge for infection control teams? J. Hosp. Infect. 72, 269–271. 10.1016/j.jhin.2009.04.002 19481297

[B23] Moodley A. Nielsen S. S. Guardabassi L. (2011). Effects of tetracycline and zinc on selection of methicillin-resistant *Staphylococcus aureus* (MRSA) sequence type 398 in pigs. Vet. Microbiol. 152, 420–423. 10.1016/j.vetmic.2011.05.025 21664077

[B24] Nemeghaire S. Vanderhaeghen W. Argudín M. A. Haesebrouck F. Butaye P. (2014). Characterization of methicillin-resistant *Staphylococcus sciuri* isolates from industrially raised pigs, cattle and broiler chickens. J. Antimicrob. Chemother 69, 2928–2934. 10.1093/jac/dku268 25063778

[B25] Paterson G. K. Morgan F. J. Harrison E. M. Peacock S. J. Parkhill J. Zadoks R. N.. (2014). Prevalence and properties of mecC methicillin-resistant *Staphylococcus aureus* (MRSA) in bovine bulk tank milk in Great Britain. J. Antimicrob. Chemother 69, 598–602. 10.1093/jac/dkt417 24155057 PMC3922150

[B26] Peeters L. E. Argudín M. A. Azadikhah S. Butaye P. (2015). Antimicrobial resistance and population structure of *Staphylococcus aureus* recovered from pigs farms. Vet. Microbiol. 180, 151–156. 10.1016/j.vetmic.2015.08.018 26350798

[B27] Piccinini R. Tassi R. Daprà V. Pilla R. Fenner J. Carter B.. (2012). Study of *Staphylococcus aureus* collected at slaughter from dairy cows with chronic mastitis. J. Dairy Res. 79, 249–255. 10.1017/S002202991200009X 22369758

[B28] Pletinckx L. J. Verhegghe M. Crombé F. Dewulf J. De Bleecker Y. Rasschaert G.. (2013). Evidence of possible methicillin-resistant *Staphylococcus aureus* ST398 spread between pigs and other animals and people residing on the same farm. Prev. Vet. Med. 109, 293–303. 10.1016/j.prevetmed.2012.10.019 23200313

[B29] Price L. B. Stegger M. Hasman H. Aziz M. Larsen J. Andersen P. S.. (2012). *Staphylococcus aureus* CC398: host adaptation and emergence of methicillin resistance in livestock. mBio 3:e00305-11. 10.1128/mBio.00305-11 22354957 PMC3280451

[B30] Stamatakis A. (2014). RAxML version 8: a tool for phylogenetic analysis and post-analysis of large phylogenies. Bioinformatics 30, 1312–1313. 10.1093/bioinformatics/btu033 24451623 PMC3998144

[B31] Stefani S. Chung D. R. Lindsay J. A. Friedrich A. W. Kearns A. M. Westh H.. (2012). Meticillin-resistant *Staphylococcus aureus* (MRSA): global epidemiology and harmonisation of typing methods. Int. J. Antimicrob. Agents 39, 273–282. 10.1016/j.ijantimicag.2011.09.030 22230333

[B32] Stegger M. Liu C. M. Larsen J. Soldanova K. Aziz M. Contente-Cuomo T.. (2013). Rapid differentiation between livestock-associated and livestock-independent *Staphylococcus aureus* CC398 clades. PLoS ONE 8:e79645. 10.1371/journal.pone.0079645 24244535 PMC3828327

[B33] Tong S. Y. Holden M. T. Nickerson E. K. Cooper B. S. Köser C. U. Cori A.. (2015). Genome sequencing defines phylogeny and spread of methicillin-resistant *Staphylococcus aureus* in a high transmission setting. Genome Res. 25, 111–118. 10.1101/gr.174730.114 25491771 PMC4317166

[B34] Vandendriessche S. Vanderhaeghen W. Larsen J. de Mendonça R. Hallin M. Butaye P.. (2014). High genetic diversity of methicillin-susceptible *Staphylococcus aureus* (MSSA) from humans and animals on livestock farms and presence of SCCmec remnant DNA in MSSA CC398. J. Antimicrob. Chemother 69, 355–362. 10.1093/jac/dkt366 24072172

[B35] Voss A. Loeffen F. Bakker J. Klaassen C. Wulf M. (2005). Methicillin-resistant *Staphylococcus aureus* in pig farming. Emerg. Infect. Dis. 11, 1965–1966. 10.3201/eid1112.050428 16485492 PMC3367632

[B36] Ward M. J. Gibbons C. L. McAdam P. R. van Bunnik B. A. Girvan E. K. Edwards G. F.. (2014). Time-scaled evolutionary analysis of the transmission and antibiotic resistance dynamics of *Staphylococcus aureus* Clonal Complex 398. Appl. Environ. Microbiol. 80, 7275–7282. 10.1128/AEM.01777-14 25239891 PMC4249192

